# Modifications in stromal extracellular matrix of aged corneas can be induced by ultraviolet A irradiation

**DOI:** 10.1111/acel.12324

**Published:** 2015-02-25

**Authors:** Sébastien P Gendron, Patrick J Rochette

**Affiliations:** 1 Centre de Recherche du CHU de Québec, Axe Médecine Régénératrice, Hôpital du Saint-SacrementQuébec, QC, Canada; 2 Centre de Recherche en Organogenèse Expérimentale de l'Université Laval/LOEXQuébec, QC, Canada; 3 Département d'Ophtalmologie, Faculté de Médecine, Université LavalQuébec, QC, Canada

**Keywords:** Ultraviolet A, photoaging, corneal stroma, extracellular matrix, collagen, proteoglycan, environmental toxicity

## Abstract

With age, structural and functional changes can be observed in human cornea. Some studies have shown a loss of corneal transparency and an increase in turbidity associated with aging. These changes are caused by modifications in the composition and arrangement of extracellular matrix in the corneal stroma. In human skin, it is well documented that exposure to solar radiation, and mainly to the UVA wavelengths, leads to phenotypes of photoaging characterized by alteration in extracellular matrix of the dermis. Although the cornea is also exposed to solar radiation, the extracellular matrix modifications observed in aging corneas have been mainly attributed to chronological aging and not to solar exposure. To ascertain the real implication of UVA exposure in extracellular matrix changes observed with age in human cornea, we have developed a model of photoaging by chronically exposing corneal stroma keratocytes with a precise UVA irradiation protocol. Using this model, we have analyzed UVA-induced transcriptomic and proteomic changes in corneal stroma. Our results show that cumulative UVA exposure causes changes in extracellular matrix that are found in corneal stromas of aged individuals, suggesting that solar exposure catalyzes corneal aging. Indeed, we observe a downregulation of collagen and proteoglycan gene expression and a reduction in proteoglycan production and secretion in response to cumulative UVA exposure. This study provides the first evidence that chronic ocular exposure to sunlight affects extracellular matrix composition and thus plays a role in corneal changes observed with age.

## Introduction

The cornea plays a protective role by blocking the majority of ultraviolet (UV; 100–400 nm) while allowing visible light (400–750 nm) to reach the posterior part of the eye. The cornea is the principal refractive element in the eye. It is composed of 3 cellular layers: epithelium, stroma, and endothelium. The epithelium, the most anterior corneal layer, contains 6–8 strata of epithelial cells. Underlying the epithelium, the stroma represents up to 90% of the corneal thickness. It confers the biomechanical and optical properties to the cornea and is mainly composed of specifically oriented collagen fibers and a few dispersed extracellular matrix-producing keratocytes (Ruberti *et al*., 2011). The endothelium is the most posterior layer of the cornea and is composed of a monolayer of endothelial cells. It maintains the corneal stroma in a relatively dehydrated state by creating a semipermeable barrier between the cornea and the anterior ocular chamber (Bonanno, 2011).

Light transmission in human corneas from patients of different ages is documented. It was first measured in 1962 by Boettner and Wolter in corneas from nine patients ranging from 4 weeks to 75 years of age (Boettner & Wolter, 1962). Their results showed a loss of corneal transparency with age. In 1980, Lerman confirmed the age-dependent opacification found by Boettner and Wolter by measuring corneal transparency in patients of three different ages (8, 24, and 80 years old) (Lerman, 1980). Beems and van Best, in 1990, have measured corneal transmission in eight patients between 22 and 87 years old. They found a slight but nonsignificant age-dependent loss of corneal transparency (Beems & Van Best, 1990). Although there is some evidence that aging affects corneal transparency, this remains a subject of controversy.

Despite the controversy resulting from data on the measurement of corneal transparency in aging corneas, it is well understood that important changes occur with age in the corneal stroma extracellular matrix (ECM). An increased turbidity (Stocker & Moore, 1975) and stromal density (Weale, 1963) can be observed clinically. A change in the collagen fiber spacing and in degeneration, providing areas free of collagen, is observed with aging (Kanai & Kaufman, 1973). An increase in glycation and in advanced glycation end products (AGEs) is also associated with age (Malik *et al*., 1992). It has also been shown that the corneal collagen interfibrillar spacing is decreased with age, and this may be related to changes in the interfibrillar matrix proteoglycan composition (Malik *et al*., 1992; Malik & Meek, 1996; Daxer *et al*., 1998). Moreover, considerable stiffening is observed with age, possibly related to the age-related nonenzymatic cross-linking of the corneal stroma collagen fibrils (Elsheikh *et al*., 2007, 2010). Most of the studies on corneal changes are observational and none have precisely analyzed the extent of ECM changes with age.

In humans, the skin and the eye are the only two organs exposed to UV radiation from the sun. The photoaging-induced biochemical and molecular changes in dermal ECM are well defined. They include the aberrant overproduction of elastin, known as solar elastosis (Bernstein *et al*., 1995; Bernstein & Uitto, 1996). Cumulative UV exposure also affects dermal collagens, the most abundant dermal ECM proteins, representing 70% of the dry skin mass (Gniadecka *et al*., 1998). There is strong evidence showing that photoaging leads to a decrease in collagen synthesis (Fisher *et al*., 2000; Quan *et al*., 2004). Moreover, the reactive oxygen species induced by UV radiation stimulate the matrix metalloproteinase (MMP) gene expression through the MAPK signaling pathways (Pimienta & Pascual, 2007; Yaar & Gilchrest, 2007). MMPs are responsible for the collagen degradation observed in photoaged skin (Fisher *et al*., 2002; Quan *et al*., 2009). These photoaging-related changes in the skin are primarily attributed to the UVA component of UV light (Krutmann, 2000). UVA rays penetrate deeper in the dermis and induce reactive oxygen species orders of magnitude more efficiently than UVB via chromophore photosensitization reactions (Baier *et al*., 2006; Tewari *et al*., 2012).

Similar to the skin, the cornea is constantly exposed to sun's UV radiation, and we have shown that UVA penetrates throughout the corneal stroma (Mallet & Rochette, 2011, 2013). However, the modifications observed in the ECM of aging corneas have been mainly attributed to chronological aging, ignoring the real implication of UV light in those changes. To our knowledge, the biochemical and molecular consequences of cumulative corneal UV exposure have not been documented. This is mainly attributed to a lack of a proper model to study. In skin, it is possible to compare regions of sun-exposed and sun-protected skin in the same individual, allowing the precise determination of photo-induced changes in skin. This strategy is not possible for studies of the effect of cumulative UV exposure in the cornea. Therefore, we have developed a model of corneal photoaging to study the effects of cumulative UV exposure in the cornea. Corneal stroma keratocytes have been chronically UVA-irradiated, and the transcriptome and the proteome have been analyzed. Our results clearly show a downregulation of collagen and proteoglycan gene expression and a reduction in proteoglycan production and secretion in response to cumulative UVA exposure. This study provides the first evidence that chronic ocular exposure to sunlight affects ECM composition and thus plays a role in corneal changes observed with age.

## Materials and methods

All experiments performed in this study were conducted in accordance with our institution's guidelines and the Declaration of Helsinki. The research protocols received approval by the CHU de Québec institutional committee for the protection of human subjects.

### Cell culture

Corneal stroma keratocytes were obtained from the eyes of 6-day-old and 87-year-old human subjects (provided by La Banque d'Yeux du Centre Universitaire d'Ophtalmologie, Québec, Canada). Cells were cultured to full confluence in DMEM medium (Wisent, St-Bruno, Québec, Canada) with 10% FBS (Wisent, St-Bruno, Québec, Canada) and 1% penicillin/streptomycin (Wisent, St-Bruno, Québec, Canada) at 37 °C, 5% CO_2_. Cells were used between passages 1 and 3.

### UVA irradiation

**Fig 1:**
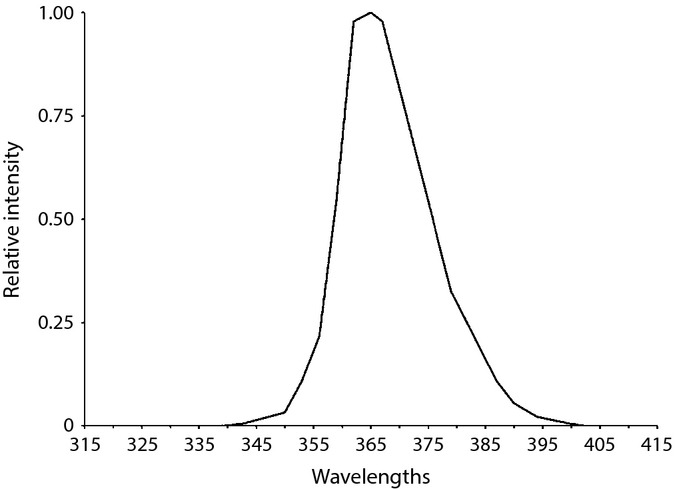
Emission spectrum of the UVA lamp. UVA irradiations were performed using B100 (UVP) lamps. The spectrum is derived from the manufacturer's specifications and modified according to measurements made using an International Light double monochromator spectroradiometer (IL7000/760D/790).

Fully confluent keratocytes of a 6-day-old patient were irradiated with a UVA lamp emitting UVA1 wavelengths (340–400 nm) with < 0.01% of UVA2 wavelengths (315–340 nm) (Fig.1) (UVP 365-nm lamp with filter, Upland, CA, USA). Cells were irradiated with 20 kJ m^−2^ UVA, two times a day, 5 days a week for 9 weeks (90 × 20 kJ m^−2^, for a total of 1800 kJ m^−2^ UVA). During the irradiation, DMEM medium was changed to PBS (Wisent, St-Bruno, Québec, Canada) to avoid oxidative stress coming from the irradiated culture medium. New medium was added after each irradiation. For the control, cells from the same strain were cultured in the same conditions but not UVA-irradiated. The medium was changed to PBS at the same frequency as the irradiated cells.

### Gene profiling

Total RNA was isolated from irradiated and nonirradiated 6-day-old corneal stroma keratocytes at 100% confluence with TRIzol reagent (Ambion; Life Technologies, Grand Island, NY, USA) using the manufacturer's protocol. Cyanine 3-CTP (Agilent protocol)-labeled cRNA targets were prepared from 200 ng of total RNA from both experimental samples using the Agilent One-color Microarray-Based Gene Expression Analysis kit (Agilent Technologies, Santa Clara, CA, USA). Then, 600 ng cRNA was incubated on a G4851A SurePrint G3 Human Ge 8 × 60 K array slide (60 000 probes; Agilent Technologies). Slides were then hybridized for 18 h, washed, and scanned on an Agilent SureScan Scanner. Data were then analyzed using the Arraystar V4.1 (DNAstar, Madison, WI, USA) software for scatter plot and heatmap generation for selected genes. Statistical analysis of data has been performed using the robust multiarray analysis for background correction of the raw data values. Microarray data shown in this study comply with the Minimum Information About a Microarray Experiment (MIAME) requirements (Brazma *et al*., 2001).

### Engineered corneal stroma production

After UVA irradiation (90 × 20 kJ m^−2^), irradiated and nonirradiated 6-day-old and 87-year-old cells were cultured for 40 days in medium containing 50 μg mL^−1^ ascorbic acid, using a protocol adapted from Proulx *et al*. (2010). Ascorbic acid allows fibroblasts to secrete and assemble ECM to form an engineered corneal stroma. The engineered stroma was then partially decellularized with 1.8 mm sodium deoxycholate, which is beneath the critical micellar concentration of 2 mm at 25 °C. This concentration avoids solubilization of the ECM in the decellularization process. Engineered stroma was washed 3 times with PBS at 37 °C and 1.8 mm sodium deoxycholate was then added to the engineered stromas for 15 min at room temperature. Stromas were then washed 5 times with PBS at 37 °C. Decellularized stromas were stored at −80 °C.

### Mass spectrometry analysis

The protein preparation and mass spectrometry analysis were performed by the Proteomics platform of the Eastern Quebec Genomic Center, CHU de Quebec, Canada. Decellularized engineered corneal stromas were sent to the platform for protein separation on gels and further processing. Each lane of the protein gel was cut into ten bands and then washed with water. Tryptic digestion was performed on a MassPrep liquid handling robot (Waters, Milford, MA, USA) according to the manufacturer's specifications and the protocol in Shevchenko *et al*. (1996) with the modifications suggested by Havlis *et al*. (2003). Briefly, proteins were reduced with 10 mm DTT and alkylated with 55 mm iodoacetamide. Trypsin digestion was performed using 126 nm of modified porcine trypsin (Sequencing grade; Promega, Madison, WI, USA) at 58 °C for 1 h. Digestion products were extracted using 1% formic acid, 2% acetonitrile followed by 1% formic acid, 50% acetonitrile. The recovered extracts were pooled, dried by vacuum centrifuge, resuspended in 10 μL of 0.1% formic acid, and analyzed by mass spectrometry.

Peptide samples were separated by online reversed-phase (RP) nanoscale capillary liquid chromatography (nanoLC) and analyzed by electrospray ionization–tandem mass spectrometry (ESI-MS/MS). The experiments were performed with an Agilent 1200 nanopump connected to a 5600 mass spectrometer (AB Sciex, Framingham, MA, USA) equipped with a nanoelectrospray ion source. Peptide separation occurred on a self-packed PicoFrit column (New Objective, Woburn, MA, USA) packed with Jupiter (Phenomenex, Torrance, CA, USA) 5u, 300A C18, 15 cm × 0.075 mm internal diameter. Peptides were eluted in a linear gradient with 2–50% solvent B (acetonitrile, 0.1% formic acid) in 30 min, at 300 nL min^−1^. Mass spectra were acquired using a data-dependent acquisition mode using Analyst software version 1.6. Each full scan mass spectrum (400–1250 m/z) was followed by collision-induced dissociation of the 20 most intense ions. Dynamic exclusion was set for a period of 3 s and a tolerance of 100 ppm.

### Database searching

All MS/MS peak lists (MGF files) were generated using ProteinPilot (AB Sciex, Version 4.5) with the Paragon algorithm. MGF peak list files were created using Protein Pilot version 4.5 software (ABSciex, Concord, Ontario, Canada) utilizing the Paragon and Progroup algorithms (Shilov). MGF sample files were then analyzed using Mascot (Matrix Science, London, UK; version 2.4.0). Mascot was searched with a fragment ion mass tolerance of 0.100 Da and a parent ion tolerance of 0.100 Da. Carbamidomethyl of cysteine was specified in Mascot as a fixed modification. Glu->pyro-Glu of the n-terminus, gln->pyro-Glu of the n-terminus, deamidation of asparagine and glutamine, and oxidation of methionine were specified in Mascot as variable modifications.

### Criteria for protein identification

Scaffold (version Scaffold 4.3.0, Proteome Software Inc., Portland, OR, USA) was used to validate MS/MS-based peptide and protein identifications. Peptide identifications were accepted if they could be established at > 95.0% probability by the Peptide Prophet algorithm (Keller *et al*., 2002; Nesvizhskii *et al*., 2003). Protein identifications were accepted if they could be established at > 99.0% probability and contained at least two identified peptides. Protein probabilities were assigned by the Protein Prophet algorithm (Nesvizhskii *et al*., 2003). Proteins that contained similar peptides and could not be differentiated based on MS/MS analysis alone were grouped to satisfy the principles of parsimony.

## Results and discussion

The accumulated sunlight exposure of an individual during its lifetime is highly variable, and it is virtually impossible with the available tools to approximate the UVA dose received in the cornea of a given subject. Nonetheless, to partially mimic the solar exposure received by a cornea during a lifetime, we have developed a precise irradiation protocol. The irradiation source consists almost entirely of UVA1 wavelengths (340–400 nm) and contains < 0.01% of UVA2 wavelengths (315–340 nm) (Fig.1). The UVA dose and irradiation frequency were calibrated to avoid cell death, and the cells were irradiated at 100% confluency to reduce the replication rate. UVA rays were used in our study because (1) they represent 95% of the UV wavelengths reaching the earth surface from the sun, (2) they penetrate deeper in the corneal stroma than UVB wavelengths, reaching the posterior portion of the cornea (Mallet & Rochette, 2011, 2013), and (3) they are the main wavelength responsible for skin photoaging (Krutmann, 2000). Stress related to the irradiation procedure is induced in the cells. For instance, the culture medium is replaced by PBS during irradiation, and the cells are removed from the 37 °C incubator during the preparation and irradiation period. Those stresses can also induce transcriptomic and proteomic changes. In our experiment, the nonirradiated controls (NoUV) underwent the same procedure as the irradiated samples (UVA). Therefore, the observed changes can safely be attributed to the UVA exposure and not to the stress caused by the procedure. Our model, using primary cultures of human corneal stroma keratocytes, also has the advantage of being fully isogenic.

**Fig 2:**
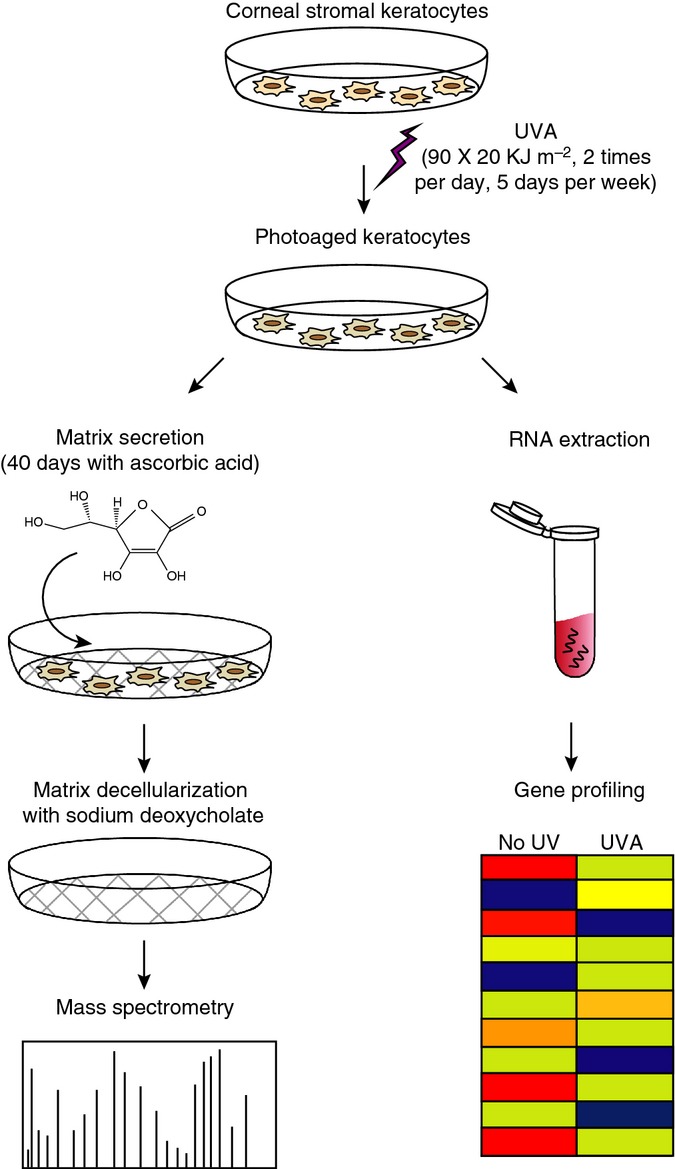
Schematic representation of the UVA irradiation protocol and further analysis of transcriptomic and proteomic changes induced by irradiation. Corneal stroma keratocytes were isolated from human corneas, cultured, and irradiated *in cellulo* using 20 kJ m^−2^ UVA, two times per day, 5 days per weeks for a total of 90 irradiations or 1800 kJ m^−2^. (1) RNA was isolated from irradiated or unirradiated control keratocytes, and the transcriptome was analyzed using microarray technology. (2) Irradiated corneal keratocytes and unirradiated controls were exposed to ascorbic acid in order to induce secretion and arrangement of extracellular matrix. This tissue engineering technique was used to reproduce corneal stroma *in vitro*. The tissue-engineered corneal stroma was decellularized using sodium deoxycholate, and the extracellular matrix (ECM) was then analyzed by mass spectrometry.

To better understand the molecular changes induced by chronic exposure to UVA, we analyzed the transcriptomic and proteomic changes induced by our irradiation protocol in human corneal keratocytes (Fig.2). For the transcriptomic analysis, RNA was isolated from artificially photoaged and control keratocytes and was analyzed using microarray technology. For the proteomic analysis, the photoaged or control stromal keratocytes were induced using ascorbic acid to secrete and arrange ECM to generate tissue-engineered corneal stromas. ECM constitution was analyzed by mass spectrometry.

### Gene profiling

**Fig 3:**
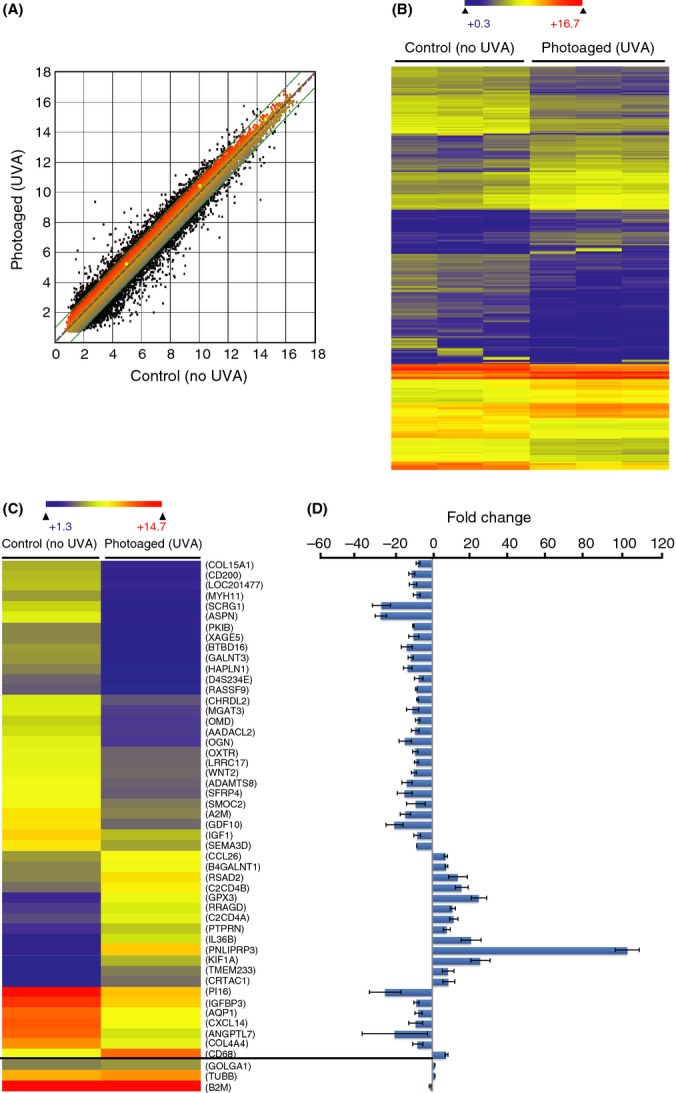
Microarray analysis of UVA-induced transcriptomic changes in human diploid stromal keratocytes. (A) Scatter plot of log2 signal intensity for 60 000 targets covering the entire human transcriptome. The signal for UVA-irradiated keratocyte targets (photoaged) (*y*-axis) is plotted against the signal of unirradiated keratocytes (control) (*x*-axis). The significantly deregulated targets (> 2-fold positively or negatively) between the two conditions are represented in black dots. (B) Heatmap depicting the significantly deregulated genes in photoaged and control keratocytes. The experiment was performed in triplicate, and the heatmap clearly shows the experimental reproducibility. The observed changes in gene expression can thus be interpreted as UVA-induced changes and not as experimental variations. The color scale used to display the log2 expression level values was determined by the hierarchical clustering algorithm of the Euclidian metric distance between genes. Genes indicated in dark blue correspond to those whose expression is very low, whereas highly expressed genes are shown in orange/red. (C) Heatmap depicting the most deregulated 48 genes by chronic UVA irradiation. Three control genes (GOLGA1, TUBB, and B2M) are used as experimental controls (bottom three genes of the heatmap). The transcription level of those genes is stable, independent of cell type and condition (Lee *et al*., 2007). Those three genes are nonsignificantly deregulated by UVA irradiation. (D) Graphical representation of the gene expression difference between photoaged and control keratocytes.

We analyzed the gene expression of artificially photoaged and control stromal keratocytes. The experiment was performed in triplicate. The transcriptome analysis of the significantly deregulated genes (Fig.3A,B) showed very little variation between each experimental triplicate, and it can be stated that the experimental procedure is reproducible.

Among the 48 most deregulated genes, some are of particular interest in regard to the sunlight-induced corneal changes (Fig.3C,D). Collagen XV (col15a1) and collagen IV (col4a4) code for two ECM proteins found in corneal stroma. They are present at a marginal level (< 1%) in corneal stroma. The exact function of collagen XV, a nonfibrillar collagen, is not well defined (Muragaki *et al*., 1994; Pihlajaniemi & Rehn, 1995). On the other hand, collagen IV is a main component of the corneal basement membrane, which interacts with corneal epithelial cells (Kurpakus Wheater *et al*., 1999). Both collagens are downregulated after irradiation. In addition, another ECM-coding gene, the proteoglycan mimecan (OGN), was highly downregulated by artificial photoaging. OGN is a keratan sulfate important in collagen fibrillogenesis and matrix assembly. Mimecan-deficient mice have thicker collagen fibrils in the corneal stroma (Pellegata *et al*., 2000).

PNLIPRP3 gene is the most upregulated gene after irradiation. This is surprising because no stress response or aging function has been reported for this gene. In fact, PNLIPRP3 and its function are not well documented, and it may be interesting to investigate its function in relation to UV-induced response. However, we consider the investigation of PNLIPRP3 to be beyond the purpose of this study.

### Collagens

**Fig 4:**
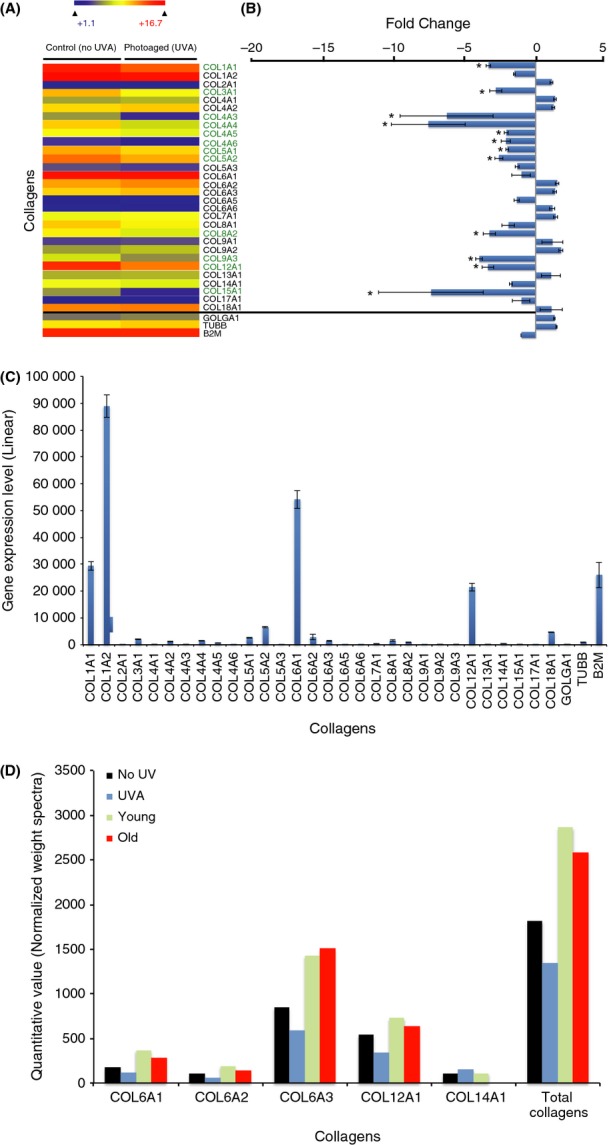
UVA-induced collagen changes in human diploid corneal keratocytes. (A) Heatmap depicting the relative expression of collagen-coding genes in photoaged and control human diploid corneal stroma keratocytes. The significant deregulated genes (>2-fold positively or negatively) between the two conditions are identified by an asterisk (*) (B) Graphical representation of collagen-coding gene expression differences between photoaged and control keratocytes. The vast majority of collagen-coding genes are downregulated in photoaged cells, and 12 are significantly downregulated. These include collagen-coding genes from the collagen I, III, IV, V, VIII, IX, XII, and XV families. (C) Linear expression level of collagen-coding genes in unirradiated control keratocytes. Collagen I-coding genes (col1a1 and col1a2) are the most expressed (approximately 60% of all collagen-coding gene expression). This correlates with the fact that collagen I represents 75% of all collagens found in corneal stroma. Collagen VI-coding genes (col6a1, a2, a3, a4, a5, and a6) and the collagen XII-coding gene (col12a1) are the second and the third most expressed (approximately 30% and 10% of all collagen-coding gene expression, respectively). (D) Graphical representation of the mass spectrometry analysis depicting collagen protein levels in photoaged keratocytes (UVA) and in unirradiated controls (NoUV). The collagen protein levels in corneal stromal keratocytes from 6-day-old (Young) and 87-year-old (Old) patients were used as a comparison. The results show a downregulation of the detected collagens in photoaged keratocytes. This tendency is not reproduced when comparing collagen secretion between keratocytes from young and old patients. Using mass spectrometry, fibril-associated collagens with interrupted triple helices (FACIT) and nonfibrillar collagens were mainly detected. Although fibrillar collagens are the main constituent of the corneal stroma, their detection by mass spectrometry is challenging.

The predominant collagens found in corneal stroma are collagen I and VI, representing 75% and 17% of total stromal ECM collagen, respectively (Michelacci, 2003). The genes encoding those collagens also have the highest expression in corneal stroma keratocytes (Fig.4A–C). The chronic UVA irradiation induces a significant downregulation of genes coding for collagen I (col1a1), collagen III (col3a1), collagen IV (col4a3, a4, a5, and a6), collagen V (col5a1 and a2), collagen VIII (col8a2), collagen IX (col9a3), collagen XII (col12a1), and collagen XV (col15a1). None of the collagen-coding genes were upregulated by the UVA irradiation regime.

With the help of ascorbic acid, the secreted collagens are cross-linked, assembled into fibrils, and aggregated into fibers (Proulx *et al*., 2010). We analyzed the assembled collagen content in the tissue-engineered corneal stromas using mass spectrometry (Fig.4D). Collagen VI (col6a1, a2, and a3), collagen XII (col12a1), and collagen XIV (col14a1) were detected by mass spectrometry. Collagen I, the predominant collagen normally found in corneal stroma, is a fibril-forming collagen. Consequently, we found it at an almost undetectable level using standard mass spectrometry procedure. Genes coding for collagen VI and XII were the most highly expressed non-fibril-forming collagens, which correlated with the fact that they are the ones detected at the highest level using mass spectrometry. A small reduction in collagen VI and XII was observed in our irradiated stromal keratocytes when compared with the unirradiated controls (Fig.4D). However, this reduction was either less important or not apparent when comparing stromal cells from an old donor (87 years old) with a young donor (6 days old) (Fig.4D). This indicates that the cumulative UVA exposure throughout a lifetime does not contribute significantly to differences in corneal stroma collagen deposition.

### Proteoglycans and other ECM

**Fig 5:**
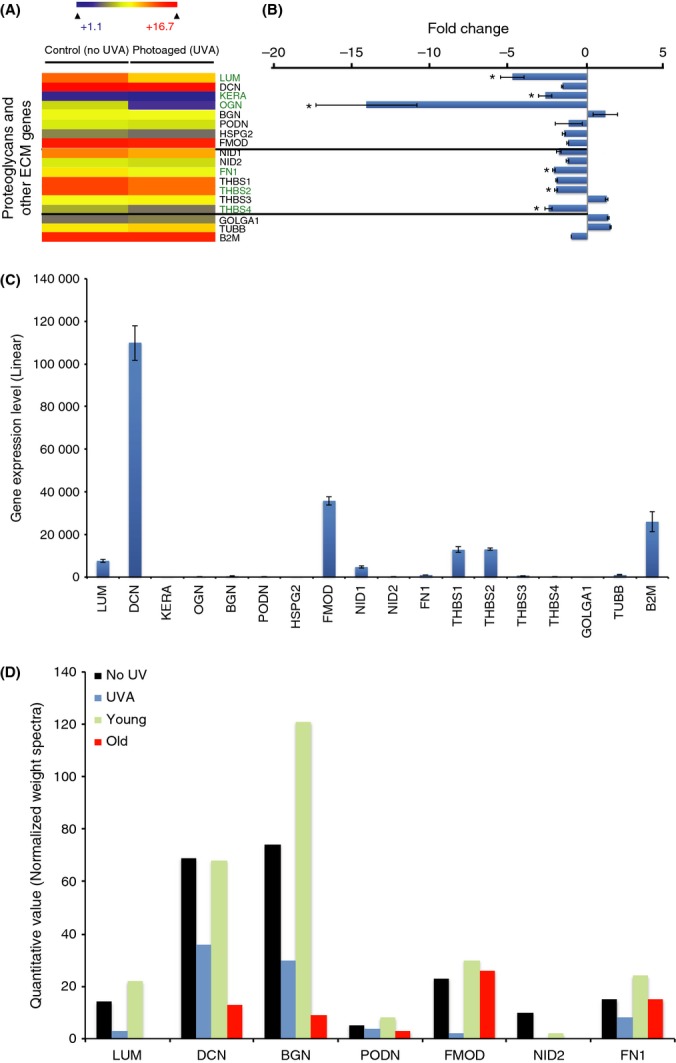
UVA-induced proteoglycans and other extracellular matrix (ECM) changes in human diploid corneal keratocytes. (A) Heatmap depicting the relative expression of proteoglycans and other ECM-coding genes in photoaged and control diploid corneal stroma keratocytes. The top eight genes (above the top horizontal line) code for proteoglycans, the middle seven genes (between the two horizontal lines) code for other ECM, and the bottom three genes are housekeeping genes used as controls. The significant deregulated genes (>2-fold positively or negatively) between the two conditions are identified by an asterisk (*) (B) Graphical representation of proteoglycan and other ECM-coding gene expression differences between photoaged and control keratocytes. Three major proteoglycan-coding genes are significantly downregulated (more than 2×) by chronic UVA irradiation (i.e., lumican, keratocan, and mimecan). Among the proteoglycan-coding genes, mimecan is the most deregulated in photoaged cells, with a > 14-fold decrease. Among the other ECM-coding genes, three are significantly downregulated by the UVA irradiation, that is, fibronectin I, thrombospondin 2 and 4. None of the proteoglycans and other ECM genes were upregulated by UVA irradiation. (C) Linear expression level of proteoglycan and other ECM-coding genes in unirradiated control keratocytes. Decorin showed the highest expression level. Among the UVA-induced dysregulated genes, lumican showed the highest expression level. (D) Graphical representation of the mass spectrometry analysis depicting proteoglycan and other ECM protein levels in photoaged keratocytes (UVA), unirradiated controls (NoUV), and keratocytes from 6-day-old (Young) and 87-year-old (Old) patients. The result shows a clear downregulation of all detected proteoglycans (lumican, decorin, biglycan, podocan, and fibromodulin) by both UVA irradiation and age. Among other ECM proteins, fibronectin, a temporary ECM component used in corneal wound healing to promote corneal epithelial cell adhesion and migration (Murakami *et al*., 1992; Kang *et al*., 1999; Tanaka *et al*., 1999), is downregulated by both UVA irradiation and age. The presence of fibronectin is an indication that our tissue-engineered stromas are not completely mature (Lake *et al*., 2013).

Proteoglycans are responsible for the organization and alignment of collagen fibers [reviewed in (Hassell & Birk, 2010)]. Their presence at a precise level is crucial for corneal transparency. For example, lumican knockout mice exhibit severe corneal opacity and corneal thinning associated with disorganized collagen fibrils in the corneal stroma (Meij *et al*., 2007). Lumican is responsible for the organization and spacing of collagen fibrils and the regulation of collagen assembly, and lumican^−/−^ mice develop bilateral corneal opacification (Chakravarti *et al*., 1998). Lumican plays a regulatory role for other keratocans, and a downregulation of lumican leads to a downregulation of keratocans such as biglycan, decorin, and podocan (Chen & Birk, 2013). Gene expression of lumican, keratocan, and biglycan (OGN) was significantly downregulated by chronic UVA irradiation (Fig.5A,B). We also observed a downregulation of decorin (DCN) and podocan (PODN) gene expression, but it is not statistically significant.

At the protein level, the mass spectrometry analysis of the engineered stromal ECM showed a clear decrease of different proteoglycans (Fig.5D). This is in accordance with the downregulation of proteoglycan gene expression observed in transcriptome analysis (Fig.5A,B). Lumican, decorin, biglycan, podocan, and fibromodulin were all found at lower levels in engineered stroma from irradiated cells when compared with unirradiated controls. The decrease in the protein levels of all proteoglycans was also observed in engineered stromas from cells from 87-year-old subjects when compared with cells from 6-day-old subjects. This clearly indicates that cumulative UVA exposure to the cornea is implicated in the depletion of corneal stroma proteoglycans observed with aging.

Among other ECM-coding genes, a significant downregulation of fibronectin (FN1) and thrombospondin (THBS2 and 4) was observed (Fig.5D). Fibronectin and thrombospondin are not found in native corneal stroma. However, they are important in corneal wound healing (Matsuba *et al*., 2011; Blanco-Mezquita *et al*., 2013). The corneal stroma keratocytes cultured *in vitro* adopt characteristics of fibroblasts, mimicking what can be observed in the corneal wound healing process (Matsuba *et al*., 2011). This explains, at least in part, the expression of those genes in cultured keratocytes. We observed the depletion in fibronectin associated with chronic UVA exposure in engineered stromas, and this depletion was also observed in 87-year-old cells (Fig.5D). However, because fibronectin is associated with wound healing, this may reflect that UVA influences wound healing rather than corneal stroma composition. The gene encoding nidogen 2 (NID2) is downregulated by UVA, and its protein level is also depleted to a level undetectable by mass spectrometry in both engineered stromas from irradiated keratocytes (UVA) and keratocytes from a 87-year-old subject (Old) (Fig.5). In the native cornea, nidogen 2 is found in the basement membrane at the epithelium/stroma junction. Nidogen 2 is expressed on both sides of basement membrane in infants, but it is expressed only on the epithelial side of the membrane in adults (Kabosova *et al*., 2007). Because our model of tissue-engineered stroma does not contain an epithelium and/or a membrane, the conclusions that can be drawn from this result are limited.

### Matrix-degrading metalloproteinases

**Fig 6:**
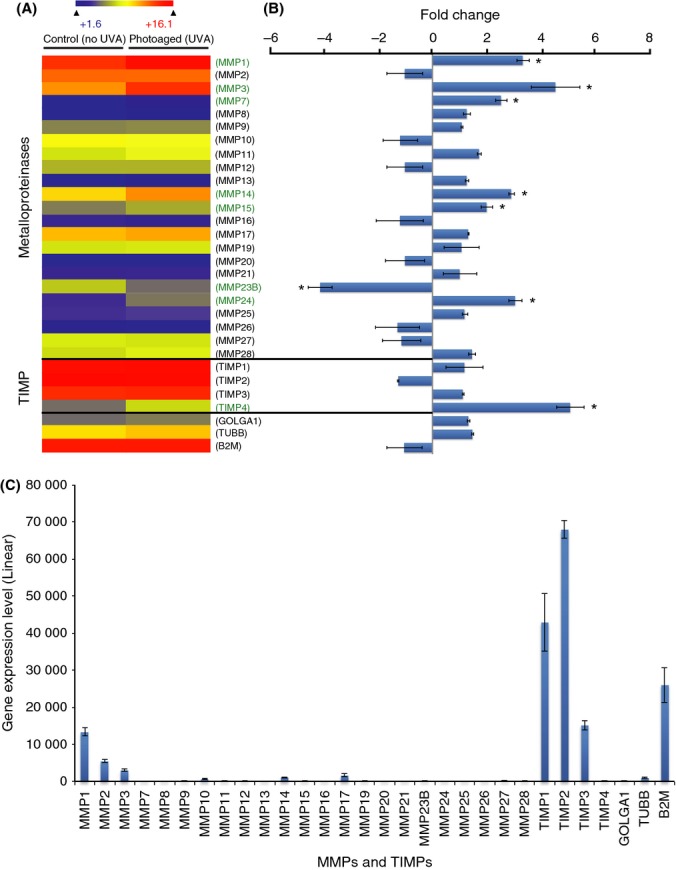
UVA-induced metalloproteinase (MMP) and TIMP changes in human diploid corneal keratocytes. (A) Heatmap depicting the relative expression of MMP- and TIMP-coding genes in photoaged and control diploid corneal stroma keratocytes. The significant deregulated genes (>2-fold positively or negatively) between the two conditions are identified by an asterisk (*) (B) Graphical representation of MMP- and TIMP-coding gene expression differences between photoaged and control keratocytes. A general upregulation of MMP-coding genes caused by UVA irradiation was observed. More precisely, six MMP-coding genes (MMP1, 3, 7, 14, 15, and 24) were significantly upregulated by UVA irradiation and only one (MMP23B) was downregulated. On the other hand, only TIMP4 is upregulated and no TIMP family member is downregulated by the UVA irradiation. (C) Linear expression level of MMP- and TIMP-coding genes in unirradiated control keratocytes. MMP1, 2, and 3 are the most highly expressed MMPs, but their expression levels are far from those of TIMP-coding genes (TIMP1, 2, and 3). The expression level of TIMP4, the only one found deregulated by UVA irradiation, is marginal compared to the other TIMPs.

We then analyzed gene expression of matrix-degrading MMPs and MMP inhibitors (TIMP) (Fig.6). MMPs are proteinases responsible for the degradation of ECM proteins, including collagens and proteoglycans (Nelson *et al*., 2000; Sternlicht & Werb, 2001). The role of MMPs in skin photoaging is well defined [reviewed in (Fisher *et al*., 2002; Quan *et al*., 2009)]. UV exposure leads to a strong increase in the expression of the genes coding for MMP1 and MMP3 and a modest increase in MMP9 in human skin *in vivo* (Fisher *et al*., 1996, 1997, 2002; Fisher & Voorhees, 1998; Brenneisen *et al*., 2002; Hazane *et al*., 2005; Wang *et al*., 2008). In agreement with findings in skin, expression of MMP1 and MMP3 increased in corneal stroma keratocytes. In total, 6 MMP-coding genes were significantly upregulated (MMP1, 3, 7, 14, 15, and 24) (Fig.6A,B). This result validates our model of corneal photoaging and supports our hypothesis that the accumulation of UVA irradiation in the eye produces effects through a process similar to skin photoaging. Only one MMP-coding gene, MMP23B, was downregulated by UVA irradiation. MMP23B is predominantly expressed in reproductive tissues, and no role in corneal ECM has been attributed to this MMP (Velasco *et al*., 1999; Ohnishi *et al*., 2001). On the other hand, among the TIMPs, only the TIMP4-coding gene is significantly upregulated by the irradiation protocol. TIMP4 has a central role in MMP regulation. It inhibits MMP1, 2, 3, 7, and 9 [reviewed in (Melendez-Zajgla *et al*., 2008)]. However, the relative expression level of TIMP4 is marginal compared to the other 3 TIMPs (Fig.6C). The imbalance of the MMP/TIMP ratio is responsible for the ECM degradation observed in skin photoaging (Hachiya *et al*., 2009). Based on our results, it would be hazardous to draw any conclusions about the potential role of MMPs or the inhibition of their effect by TIMPs in the UVA-induced ECM changes observed in corneal stroma keratocytes.

## Conclusion

Our study focuses on two aspects: (i) the development of a model of corneal photoaging and (ii) the molecular characterization of corneal photoaging. We developed a specific irradiation protocol to accumulate large amounts of UVA (1800 kJ m^−2^) in corneal stroma keratocytes without inducing significant mortality. Considering that we are exposed to approximately 50 kJ m^−2^ per h at the zenith of summer (Kuluncsics *et al*., 1999), we exposed our experimental stromal keratocytes to the equivalent of 36 h of direct sunlight. However, because many factors reduce the sun exposure received by our stromal cells, the actual time spent outside to reach 1800 kJ m^−2^ of UVA wavelengths in our keratocytes is much higher than the given 36 h. First, the sun intensity varies and is rarely at its maximal intensity. Moreover, we rarely look directly at the sun, and 40% the UVA wavelengths that reach the cornea are filtered by the corneal epithelium and never reach the corneal stroma (Mallet & Rochette, 2013). Thus, even if it is impossible to estimate the average solar exposure received by an individual's corneal stroma, we can safely state that 1800 kJ m^−2^ UVA is a high dose that should suffice to induce a photoaging effect in corneal stroma cells. By comparing corneal stroma keratocytes exposed to our irradiation protocol with the same unexposed cells, we established a perfect isogenic system to study the effect of sun exposure on ECM components. This model is greatly enhanced by the fact that using tissue engineering techniques, we reproduced an artificial corneal stroma by inducing irradiated and unirradiated keratocytes to secrete and organize their ECM. Using this corneal photoaging model, we have shed light on important changes in ECM production (at the RNA level), and secretion and organization (at the protein level) induced by UVA light. We have shown that proteoglycans are highly affected by chronic UVA irradiation, and this deregulation is also found in the corneal stromas of older subjects. These results strongly suggest that UVA plays an important role in corneal aging and that the phenotypes of corneal aging could be prevented using appropriate eye protection against UVA wavelengths. This work is an important first step in the understanding of corneal photoaging. It is well accepted that UVA exerts their biological effects mostly via the generation of reactive oxygen species (Cadet & Douki, 2011). In the perspective of this study, it would be of great interest to assess the implication of UVA-induced oxidation and photosensitizer involved in the ECM changes observed. We anticipate that with the establishment of our corneal photoaging model, more work will be conducted to understand the real impact of chronic exposure to sunlight in the human cornea.
